# Preliminary evaluation of MRI-derived input function for quantitative measurement of glucose metabolism in an integrated PET-MRI

**DOI:** 10.1186/2197-7364-2-S1-A80

**Published:** 2015-05-18

**Authors:** Udunna Anazodo, Matthew Kewin, Elizabeth Finger, Jonathan Thiessen, Jennifer Hadway, John Butler, William Pavlosky, Frank Prato, Terry Thompson, Keith St Lawrence

**Affiliations:** Lawson Health Research Institute, Department of Medical Biophysics, Western University, London, Ontario Canada; Department of Clinical Neurological Sciences, Western University, London, Ontario Canada; Diagnostic Imaging, St Joseph's Health Care, London, Ontario Canada

PET semi-quantitative methods such as relative uptake value can be robust but offer no biological information and do not account for intra-subject variability in tracer administration or clearance. Simultaneous multimodal measurements that combine PET and MRI not only permit crucial multiparametric measurements, it provides means of applying tracer kinetic modelling without the need for serial arterial blood sampling. In this study we adapted an image-derived input function (IDIF) method to improve characterization of glucose metabolism in an ongoing dementia study. Here we present preliminary results in a small group of frontotemporal dementia patients and controls. IDIF was obtained directly from dynamic PET data guided by regions of interest drawn on carotid vessels on high resolution T1-weighted MR Images. IDIF was corrected for contamination of non-arterial voxels. A validation of the method was performed in a porcine model in a PET-CT scanner comparing IDIF to direct arterial blood samples. Metabolic rate of glucose (CMRglc) was measured voxel-by-voxel in gray matter producing maps that were compared between groups. Net influx rate (Ki) and global mean CMRglc are reported. A good correlation (r = 0.9 p<0.0001) was found between corrected IDIF and input function measured from direct arterial blood sampling in the validation study. In 3 FTD and 3 controls, a trend towards hypometabolism was found in frontal, temporal and parietal lobes similar to significant differences previously reported by other groups. The global mean CMRglc and Ki observed in control subjects are in line with previous reports. In general, kinetic modelling of PET-FDG using an MR-IDIF can improve characterization of glucose metabolism in dementia. This method is feasible in multimodal studies that aim to combine PET molecular imaging with MRI as dynamic PET can be acquired along with multiple MRI measurements.

